# Involvement of Medical Students During the Coronavirus Disease 2019 Pandemic: A Cross-Sectional Survey Study

**DOI:** 10.7759/cureus.10147

**Published:** 2020-08-30

**Authors:** Richard Drexler, Jan M Hambrecht, Karl J Oldhafer

**Affiliations:** 1 Neurological Surgery, Semmelweis University, Hamburg, DEU; 2 Surgery, Semmelweis University, Hamburg, DEU

**Keywords:** covid, medical student, pandemic, education, student, covid-19, medical education, medical school, volunteer work

## Abstract

Background: The coronavirus disease 2019 (COVID-19) pandemic affects the education of medical students around the world and countries have had differing responses in dealing with this dynamic situation. The role of medical students in fighting this pandemic is controversial and it is yet to be elucidated how they can best be of service. The aim of this study is to evaluate the working fields of volunteering students and the impact of the pandemic on final year students from a student's perspective.

Methods: An anonymous online survey was conducted amongst 219 medical students from Hamburg (Germany), using an institutional online data collection program.

Results: A total of 137 questionnaires (63.5%) were completed. Of these, 97 participants were students from academic year three to five (70.8%) and 40 students were in the final year of medical school (29.2%). Of the 97 students from academic year three to five, 68 students (70.1%) signed up for voluntary duties during the pandemic. Interestingly, only 25.0% of the students were called for voluntary work in hospitals or health authorities. Final year students had already been working in hospitals since before the outbreak, with 35.0% of them assisting doctors in the treatment of COVID-19 positive patients during their placements. Using a 5-Point Likert Scale, the students who volunteered self-assessed their work as more useful and received more gratitude than final year students (p<0.01).

Conclusions: The majority of medical students are willing to make a significant contribution in the response to COVID-19 and do not wish to be overlooked. Furthermore, the current pandemic offers novel educational opportunities for medical students.

## Introduction

The coronavirus disease 2019 (COVID-19) pandemic has spread globally with major outbreaks in the USA, Italy and Spain. Although the end is not yet in sight and the final lasting impact of COVID-19 is difficult to gauge, it is clear that the current situation is overwhelming for health care systems and economies around the world. As the pandemic progresses, staff shortages will likely occur, and this raises the question of who will step up to the plate in this ongoing crisis. In this context, the role of medical students is unclear and contentious [1-2]. Due to legal requirements, social distancing was implemented and in-person medial classes have been cancelled and replaced with online lectures and virtual teaching for most medical students [3]. However, the educational situation is crucial for final year students, as they are completing clinical clerkships. These students are normally integrated into medical teams as well as clinical routines and gaining clinical experience is a crucial aspect for success in future residencies. Nevertheless, student participation in clinical care varies across medical schools and different countries. Some countries, such as Italy, China and the United Kingdom, integrated the medical students into their health care systems or graduated students early [4-6]. On the contrary, other countries cancelled clerkships and restricted patient contact for medical students [7-8]. In Germany the treatment of final year students varied across hospitals, but non-final year students were encouraged by the German Minister of Health to volunteer in healthcare facilities [9]. It is clear that there is a dissonance between medical students' roles during the COVID-19 pandemic and the progression of patient-centered medical education.

In this study, we present medical students’ involvement during the COVID-19 pandemic. We evaluate the usefulness of volunteering students as a response to the pandemic as well as the assessment from a student's perspective during volunteering. In addition, we describe the impact of the pandemic on final year students and their clinical education.

## Materials and methods

Online survey

An online survey was designed and sent to 219 medical students enrolled at the Campus Hamburg (Germany) of the Semmelweis University Budapest (Hungary) via personalised institutional email. The survey was conducted using an institutional online data collection program. Two surveys were performed, one survey for medical students from academic year three to year five and a separate survey for final sixth-year students. The surveys consisted of single, multiple-choice, and 5-Point Likert Scale questions and recruitment began on May 12, 2020. The survey was open from May 12, 2020, to May 17, 2020, and available in German. Inclusion criteria were students currently active from academic year three to six at the medical faculty Hamburg of the Semmelweis University Budapest. Ten external students piloted the survey prior to publication, and minor amendments to wording to improve clarity were made. After a period of data collection, three researchers checked the database for errors and false data independently from each other. Four main topics were addressed: (1) reasons for volunteering or rejection of volunteering; (2) work circumstances of volunteering students; (3) critical self-reflection of usefulness of volunteering and (4) impact of COVID-19 pandemic on planned clerkships and future plans after graduation. Students from academic year three to five were initially asked if they had registered as volunteers. Individuals who volunteered until May 12, 2020, then answered a more detailed questionnaire about reasons, working field, type of activity, working hours, and contact to COVID-19-positive patients. Furthermore, they self-reflected their volunteer work answering a 5-Point Likert Scale ("strongly disagree", "disagree", "neutral", "agree", "strongly agree"). The final year students were asked about the changing workload since the beginning of the COVID-19 pandemic, contact to COVID-19 positive patients, possible infection, and the impact on future residencies. In closing, final year students self-reflected their clerkship answering the 5-Point Likert Scale as described above.

Ethical approval

All study procedures were reviewed and approved by the Ethics Committee Hamburg, Germany (WF-098/20). No patient data was included. We informed participants that their answers would be anonymously used for statistical analyses and that they would not be transmitted to third parties. 

Statistical analysis

Variables were processed and analysed using IBM SPSS Statistics for Mac version 25 (IBM Corp., Armonk, NY, USA). Data were reported as number with percentage. Differences in proportions were analysed with the chi-square test or Fisher exact test. Differences in 5-Point Likert Scale between third to fifth year and final year students were compared, using the two-sample t-test as stated by Norman [10]. A two-sided p-value less than 0.05 was considered as statistically significant.

## Results

Participants

The survey was sent to 219 medical students including 58 third-year students (26.5%), 60 fourth-year students (27.4%), and 57 fifth-year students (26.0%). The remaining 44 students (20.1%) were in the final year of medical school. In total, 137 questionnaires (63.5%) were completed. Of these, 97 participants were non-final year students (70.8%) and 40 students were in final year (29.2%). 

Third- to fifth-year students

We enrolled 97 students from academic year three to five with an equal distribution within the years (p=0.78, Table 1). These students were affected by the cancellation of in-person medical classes, which were consequently replaced by online lectures. The students were therefore enabled to volunteer in hospitals or aid health authorities during the COVID-19 pandemic. Of the 97 students, the majority (70.1%) registered as volunteers, while 29 students (29.9%) did not (Table 1). Depending on their first answer, students were asked what motivated them to sign up as volunteers or to decline the opportunity. The volunteering students had quite similar answers with the majority indicating a sense of duty to society (64.7%), interest in medical activity (57.4%), and social commitment (69.1%) as their incentives. The main reason given for not signing up was a lack of time due to studying commitments or part-time jobs (55.2%). However, six students (20.7%) were either part of a high-risk group or had a first-degree family member at risk. Finally, 11 students (11.3%) received notification that their clinical clerkships were cancelled due to the on-going pandemic.

**Table 1:** Comparison between volunteers and non-volunteers. ^1^ multiple answers were possible, in total: 35 answers. ^2^ multiple answers were possible, in total: 154 answers.

Feature	Signed up for voluntary commitment		P value
	No (n=29)	Yes (n=68)	
Academic year, n (%)			
3^rd^ year	8 (27.6)	24 (35.3)	0.78
4^th^ year	11 (37.9)	19 (27.9)	
5^th^ year	10 (34.5)	25 (36.8)	
Reasons for not volunteering, n (%) ^1^			
No time	16 (55.2)	-	-
No motivation	1 (3.4)		
Lack of skills	4 (13.8)		
Already employed	5 (17.2)		
Fear of infection	3 (10.3)		
Risk group	6 (20.7)		
Reasons for volunteering, n (%) ^2^			
Sense of duty	-	44 (64.7)	-
Interest in medical activity		39 (57.4)	
Social commitment		47 (69.1)	
Improvement of skills		24 (35.3)	
Cancellation of clerkship due to Covid-19, n (%)			
Yes	4 (13.8)	7 (10.3)	0.62
No	25 (86.2)	61 (89.7)	

Volunteering students

As previously stated, 68 students signed up for voluntary commitment but only 17 students (25.0%) were called for work. These 17 students were asked further questions regarding their work circumstances in the survey (Table 2). The majority (64.7%) were called within a week after signing up. Almost half of the students (47.1%) were assisting health authority bodies, which involved telephone consultations or data administration (29.4%). Five students (29.4%) volunteered in the hospital, either on the ward, in ICU, or in the emergency department. The remaining volunteers worked in the ambulance service (11.8%) or worked in general practices (11.8%). During their voluntary work, six students (35.3%) had physical contact with patients who tested positive for COVID-19. However, none of these students were suspected of having or tested positive for COVID-19.

**Table 2:** Work circumstances of volunteering students after assignment.

Feature	Volunteering, working students (n=17)
Days from enrollment until assignment, n (%)	
≤7 days	11 (64.7)
8-14 days	3 (17.6)
>15days	3 (17.6)
Workplace, n (%)	
Ward / ICU	3 (17.6)
Emergency department	2 (11.8)
Ambulance service	2 (11.8)
Doctors office	2 (11.8)
Health authority	8 (47.1)
Type of activity, n (%)	
Support of doctors	9 (52.9)
Nursing care	3 (17.6)
Hotline service / Data administration	5 (29.4)
Weekly working hours, n (%)	
≤20 h	7 (41.2)
20-30h	6 (35.3)
>30h	4 (23.5)
Physical contact to COVID-19 patients, n (%)	
No	10 (58.8)
Yes	6 (35.3)
Unknown	1 (5.9)
COVID-19 infection, n (%)	
No	17 (100.0)

**Figure 1:**
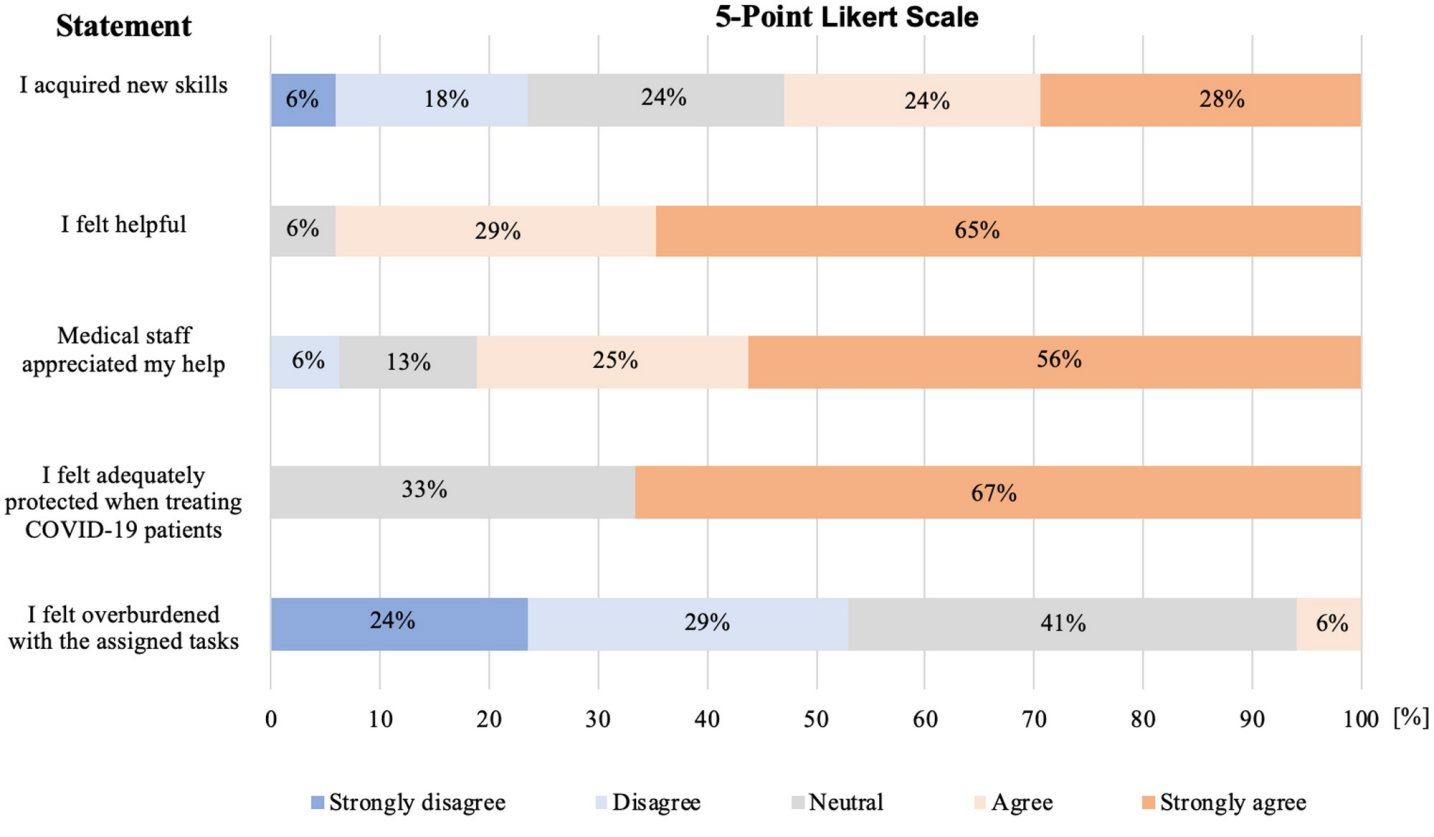
5-Point Likert Scale of medical students from academic year three to five.

To evaluate the usefulness and gratitude of the voluntary work during COVID-19 pandemic, the 17 volunteering students were confronted with several statements that were answered using a 5-Point Likert Scale (Figure 1). When self-reflecting on their work, 94% of the students felt helpful and 81% were under the impression that other medical staff valued their work. Approximately half of the volunteers acquired new skills through their work and only 6% felt overburdened.

Final year students

**Figure 2:**
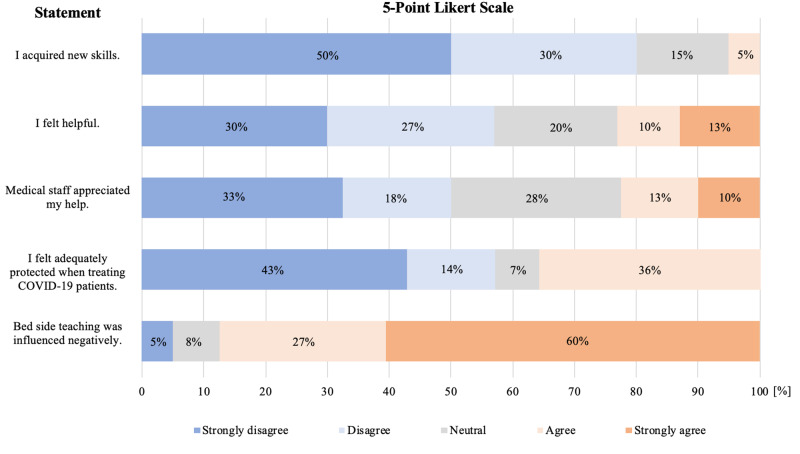
5-Point Likert Scale for final year students.

The final year students have a special status as they were already working in hospitals at the beginning of the COVID-19 pandemic. Therefore, a second survey was specifically designed to evaluate the impact of COVID-19 on their workload and clinical education. The response rate was 90.9% and consisted of 40 final year students (Table 3). When comparing the students’ workload before and during the COVID-19 pandemic, most students (72.5%) experienced a reduced workload. Of note, 14 students (35.0%) were directly involved in treating COVID-19 positive patients. During their rotations 15 students (37.5%) were suspected to be COVID-19 positive, but ultimately tested negative. As previously described for the first survey, the final year students were confronted with different statements regarding their role during the COVID-19 pandemic (Figure 2). Only 23.0% of the students evaluated their work as helpful and felt their contribution was valued by other medical staff. In addition, 5.0% agreed that they developed new skills due to the COVID-19 pandemic. Most importantly, students felt the pandemic had a negative impact on education; especially bedside teaching for example, was experienced by 87.0% of students. Focussing on future perspectives, the majority (62.5%) felt the pandemic had a negative impact on their planned residencies or research activities after graduation.

**Table 3:** Impact of the coronavirus disease 2019 (COVID-19) pandemic on final year students.

Feature	Final year students (n=40)
Workload during COVID-19 pandemic, n (%)	
Less workload	29 (72.5)
Unchanged	5 (12.5)
More workload	6 (15.0)
Physical contact to COVID-19 patients, n (%)	
No	20 (50.0)
Yes	14 (35.0)
Unknown	6 (15.0)
COVID-19 infection, n (%)	
No	25 (62.5)
Suspected	15 (37.5)
Confirmed	0 (0.0)
Cancellation of clerkship due to COVID-19, n (%)	
No	36 (90.0)
Yes	4 (10.0)
Negative impact on future residencies, n (%)	
No	15 (37.5)
Yes	25 (62.5)

Comparison between third- to fifth-year students and final year students

We described the perspectives of two student groups during the COVID-19 pandemic. On one hand, the volunteering students from academic year three to year five, and on the other, the final years in their practical placements. Both groups were equally involved in COVID-19 patient care (35.0% vs. 35.3%, p>0.05). However, final year students were more often suspected of being COVID-19 positive (p<0.01). When evaluating the role of both groups using a 5-Point Likert Scale, significant differences were observed. The volunteering students evaluated their work as more helpful and more of them felt they had acquired new skills (p<0.01). In addition, volunteers’ work was appreciated and valued more by the medical staff in comparison to final year students conducting their clinical placements (p<0.01).

## Discussion

The COVID-19 pandemic has spread around the world and poses a significant challenge ubiquitously for all health care systems. In the wake of finding solutions for possible staff shortages, the role of medical students is contentious as they could significantly contribute to the ongoing crisis. To address this eminent topic, we performed an online survey amongst medical students from Hamburg, one of Germany’s epicentres during the pandemic. We evaluated the willingness to volunteer among third to fifth-year medical students. The actual demand for students was also evaluated as well as the practicality for volunteers in hospitals and health authorities. In addition, our study focussed on the ever-changing clinical education experienced by final year students. These students were already in the final months of their practical placements when the pandemic broke out.

As the traditional didactic, in-person teaching shifted towards online lectures and virtual seminars since the beginning of the pandemic, most students wanted to contribute to the fight against the COVID-19 pandemic [2,11-14]. The results of our survey underline the willingness of medical students to contribute to the handling of the crisis as 70.1% of the students signed up for voluntary commitment. Of the remaining students who were unwilling to volunteer, nearly 40% were either already employed in a part-time job in hospitals or unable to sign up due to a higher risk of severe illness from COVID-19. However, it was remarkable that only 25.0% of the students had been called for voluntary work, mainly in the realm of hospital care or health authorities. This low number begs the question if hospitals in Germany really need student volunteers and gives the impression that hospitals are capable of solving staff shortages without the helping hands of medical students. One explanation might be that lower numbers of infections were experienced here than had been anticipated. However, when looking at current reports from several countries it becomes clear, that this abrupt conclusion cannot simply be drawn and medical students are indeed able to have a positive impact during the COVID-19 pandemic [15,16]. This is reiterated by the fact that 35% of the final year students in this study were already involved in the treatment of COVID-19 patients and the role of medical students in general could prove to be indispensible in the on-going fight against the pandemic. Furthermore, it is noteworthy that staff shortages and the demand for medical students correlates with the extent of the pandemic outbreak and the strain on the respective country’s healthcare system. As major outbreaks were experienced in Italy and the UK, medical students played a central role in dealing with the crisis in comparison to Germany [4-6]. 

However, bearing in mind the unequivocal will of medical students to participate in the fight against COVID-19, the role of medical students must be considered. Focussing on our results, volunteering students supported doctors in patient care or worked at a COVID-19 hotline service. Students who took over these tasks unburdened doctors from administrative duties so that they could entirely shift their focus to essential care of critically ill patients. In our study, volunteering students were encouraged to self-reflect on the usefulness of their tasks via a 5-Point Likert Scale. The majority of students considered their work as useful and felt it was valued by medical staff. This emphasizes that students could undertake certain tasks with a symbiotic advantage for both doctors and students, and is consistent with ideas by Miller and colleagues [2]. However, it is doubtful if these students are sufficiently trained to undertake roles with more responsibility, such as assisting with invasive ventilation of patients [17]. 

The student group most affected by the COVID-19 pandemic is undoubtedly students in their final year, due to restricted patient contact, reduced bed-side teaching and even cancelled clerkships [8,18-21]. Therefore, it is unsurprising that 87.0% of the final year students in this study reported a negative influence on their clinical education. In addition, the dissonance in suitable roles for final year students during the pandemic has lead to huge dissatisfaction in our study cohort. Final year students have gone through years of rigorous training, are nearing graduation, and therefore could have a decisive role and make a significant contribution during this pandemic. Even though clinical education is currently not the main focus, the integration of final year students into critical care of COVID-19 patients could be of indispensable value for teaching certain skills, such as critical care or ventilation therapy. Furthermore, it can prove useful for crisis management preparation with regard to possible future pandemics. The ongoing pandemic is a challenging time for maintaining clinical education for students on the cusp of graduation, but could be regarded as a worthwhile opportunity to educate them beyond the traditional curriculum. Innovative ideas are urgently needed in order to implement these novel-learning opportunities and simultaneously integrate final year students as full members into critical care or emergency teams. There have been a few promising approaches introduced in recent literature [15,16,22]. Rasmussen and colleagues initiated fast-track courses in ventilation therapy and nursing assistance for medical students attending Aalborg University and successfully integrated the majority of these students into medical care teams [15]. Another promising approach came from Klasen and colleagues who developed a training curriculum including all necessary aspects for working in a Triage-Test-Center for diagnostic swab-testing. After educating medical students according to this new curriculum, they were assigned to emergency teams that evaluated hundreds of patients daily and provided over 6700 swabs during a five-week period [16]. These concepts combine the contribution of medical students to the pandemic with the utilization of these novel learning opportunities. These newly acquired unique clinical skills further the student’s opportunities in their future career as a physician. More importantly, Rasmussen and Klasen demonstrate how medical students' role was crucial to the fight against the COVID-19 pandemic.

The involvement of medical students is entirely dependent on the gravity of the situation in the respective country. Our study showed the current situation in one of the worst-affected cities in Germany and evaluated the medical students’ involvement in the COVID-19 response from the students’ perspective. In addition, Ramussen and Klasen et al. introduced concepts functioning in Denmark and Switzerland [15-16]. It would be favourable to share further experiences from various other countries, which would show the integration of medical students into clinical settings. This could prove invaluable in the ongoing fight against COVID-19 and much can be learned from different countries’ approaches. Nevertheless, the students’ point of view must be taken into consideration in order to benefit future generations of medical students and patient care.

It is our hope that the COVID-19 pandemic leads to the development of alternative plans in how medical students could be positively utilised in future pandemics and in other exceptional circumstances, so that hospitals and medical schools are better prepared for future crises. We are convinced that pandemics, especially the current one, could offer various educational opportunities for medical students and should not lead to educational disadvantage and feelings of overall dissatisfaction.

## Conclusions

The majority of medical students are willing to make a significant contribution in the response to COVID-19 and do not wish to be overlooked. Furthermore, the current pandemic offers novel educational opportunities for medical students. However, it is mandatory that medical schools and hospitals implement functioning concepts in which students are integrated into clinical settings.
